# Microstructure and Mechanical Properties of As-Aged Mg-Zn-Sn-Mn-Al Alloys

**DOI:** 10.3390/ma16010109

**Published:** 2022-12-22

**Authors:** Caihong Hou, Zhisong Ye, Fugang Qi, Liwei Lu, Jia She, Lifei Wang, Xiaoping Ouyang, Nie Zhao, Jing Chen

**Affiliations:** 1 School of Materials Science and Engineering, Xiangtan University, Xiangtan 411105, China; 2 Hunan Provincial Key laboratory of High Efficiency and Precision Machining of Difficult-to-Cut Material, Hunan University of Science and Technology, Xiangtan 411201, China; 3 College of Materials Science and Engineering, Chongqing University, Chongqing 400045, China; 4 Shanxi Key Laboratory of Advanced Magnesium-Based Materials, Taiyuan University of Technology, Taiyuan 030024, China

**Keywords:** microstructure, mechanical properties, Mg-Zn-Sn-Mn-Al alloy, fracture mechanism, aging treatment

## Abstract

The microstructure and mechanical properties of as-aged Mg-6Zn-4Sn-1Mn-xAl (ZTM641-xAl, x = 0, 0.2, 0.5, 1, 2, 3 and 4 wt.%) alloys are studied in this paper. In terms of microstructure, the results reveal that the addition of Al mainly leads to the formation of the Al_8_Mn_5_, Al_11_Mn_4_, Al_2_Mg_5_Zn_2_ and Mg_32_(Al,Zn)_49_ phases. With increases in the addition of Al, the average grain size first decreases and then increases, while the undissolved phases increase. The average grain size of the ZTM641-0.5Al alloy is the smallest, and the single-aged and double-aged grain size is 14 μm and 12 μm, respectively. As for mechanical properties, with increases in the Al element, the strength decreases, and the elongation first increases and then decreases. The double-aged ZTM641-0.2Al alloy exhibits favorable mechanical properties at room temperature, and the UTS, YS and elongation are 384 MPa, 360 MPa and 9%, respectively. Further, the double-aged ZTM641-0.2Al alloy exhibits the comprehensive mechanical properties at 150 °C, that is, the UTS, YS and elongation are 212 MPa, 196 MPa and 29%, respectively, which is about 45% higher than that of the elongation of ZTM641. The ZTM641-xAl alloys exhibits mixed fracture at room temperature, and, with increases in the addition of Al, the fracture mechanisms of alloys are mixed fracture, ductile fracture and mixed fracture at 200 °C.

## 1. Introduction

Magnesium alloys have many excellent properties, for instance, low density, good casting performance and high recovery rate compared with other non-ferrous metals [1,2,3,4,5]. However, their development is far inferior to other non-ferrous metals due to their poor absolute strength and heat resistance [6]. Therefore, great interest has been directed towards improving the property of wrought Mg alloys [7,8].

Among various alloy systems, the typical precipitation hardening of Mg-Zn wrought alloys is widely reported. The precipitation sequence of Mg-Zn alloys during the aging state is reported to be: SSSS → GP zones → *β*_1_’-MgZn_2_ → *β*_2_’-MgZn_2_ → *β*-MgZn [9,10]. Although the rod-like *β*_1_’ phase has an effective strengthening effect, the performances of Mg-Zn alloys are still unsatisfactory [11]. At present, alloying is seen as a common way to change the microstructure and performance of alloys. The Mg-Sn alloy is also considered to be a precipitation hardening alloy, and the Mg_2_Sn phase is an excellent precipitation hardening phase [12,13]. However, since the lath-shaped Mg_2_Sn forms in the matrix and is parallel to the base plane, the precipitation hardening effect is obscure. Mendis et al. indicated that the strengthening effect of mixed Zn and Sn is better than that of single Sn or Zn, because the Zn element could improve the orientation relationship between Mg_2_Sn and matrix, resulting in a great improvement of strength [14]. As for Al, a kind of low-cost element, Oh-ishi et al. revealed that it could result in a change of the morphology and distribution of the precipitates [10]. Zhang et al. identified that the Mg-Zn-Al alloy produced three phases with Mg_32_(Al,Zn)_49_ (τ), MgZn (ε) and a ternary icosahedral structure, and the constituent was changed when the Zn and Al content changed [15]. Wan et al. studied the Mg-(8~12%)Zn-(2~6%)Al alloy, the results indicated that the creep resistance is higher when the Zn/Al is between 2 and 3, and the ZA124 alloy shows the best effects with a continuous network of τ phase structure [16]. Furthermore, recent investigations have reported that the Mn element could obviously decrease the interfacial energy of Mg-Zn alloy, then improve its strength [17]. Yin et al. found that the UTS, YS and elongation of Mg-3Zn-Mn alloy were 315.5 MPa, 275.9 MPa and 10.5%, respectively [18].

In order to better meet the needs of industrial production, the research and development of new alloys is particularly urgent. At present, there are several processing means to improve the properties of Mg alloys: One is heat treatment, namely, the common solution treatment and aging treatment [19]. The second is alloying, which is the addition of small or trace elements to Mg alloys, to change the microstructure then improve the properties of the alloy. Our team previously found that Mg-6Sn-4Zn-1Mn is an alloy with excellent comprehensive properties, and the UTS, YS and elongation of double-aged alloys are 390MPa, 378MPa and 4.16%, respectively [20]. The Al element is a good alloying element, and studies show that in an appropriate amount it can refine grain and improve the casting performance of the alloy atoms [21,22]; therefore, in this study, we developed the Mg-6Sn-4Zn-1Mn-xAl alloy, and found that the Mg-Zn-Sn-Mn-xAl alloys before heat treatment formed four new phases with the addition of the Al element, and the mechanical performances were improved [23]. To further explore the potential properties of the Mg-Zn-Sn-Mn-xAl alloy, it was heat treated with solution treatment and aging treatment. In this paper, we mainly investigate the microstructure and properties of ZTM641-xAl alloy after aging treatment.

## 2. Experimental Procedures

The experimental samples were successively melted, cast, homogenized and extrusion treatment, these specific research and experimental results are presented in this paper [16]. Subsequently, the as-extruded alloys were solution-treated (the specific parameter was heating at 430 °C for 2h) and subjected to water cooling. Then, the single-aged and double-aged alloys were treated, whose parameters were 180 °C/12 h and 90 °C/24 h + 180 °C/8 h, respectively.

The as-aged alloys were tested for ultimate tensile strength (UTS), yield strength (YS) and elongation at room temperature (150 °C and 200 °C). The test equipment was the INSTRON 3369 electronic universal material testing machine, and the strain rate was 2 mm/min. To ensure accuracy, the stress–strain curve was tested five times, and the mechanical properties of 0.2% YS, UTS and elongation were taken as the final value.

At the same time, the microstructure was also observed. The metallographic sample was observed by an Olympus BX53M optical microscope (OM). The phase composition was qualitatively analyzed by an U1tima IV X-ray diffractometer (XRD) using a Cu Kα radiation, the angle range was 10~90° and the speed was 4°/min. The phase was quantitatively analyzed by a JSM-6360 scanning electron microscope (SEM) and an Oxford INCA Energy 350 energy dispersive spectrometer (EDS). The higher resolution microstructure was analyzed by FEI Tecnai G2 F30 transmission electron microscopy (TEM) equipped with EDS detectors.

## 3. Results and Discussion

### 3.1. Microstructures of As-Aged ZTM641-xAl Alloys

**Figure 1:**
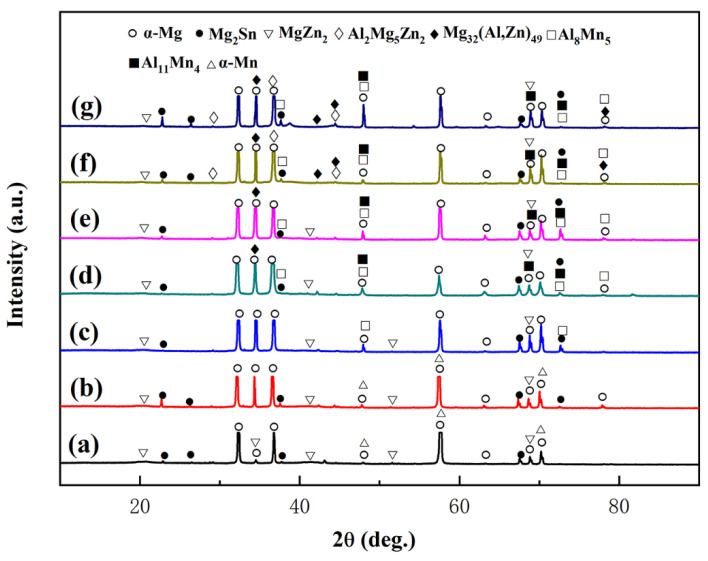
XRD patterns of the single-aged ZTM641-xAl alloys, (**a**) x = 0, (**b**) x = 0.2, (**c**) x = 0.5, (**d**) x = 1, (**e**) x = 2, (**f**) x = 3 and (**g**) x = 4.

The aging process follows the previous research results in this paper [24]: the single-aged and double-aged treatments are 180 °C/12 h and 90 °C/24 h + 180 °C/8 h, respectively. Previous studies have found that there is little difference in the phase composition between single-aged and double-aged alloys [25]. Thus, the XRD pattern of the single-aged ZTM641-xAl (x = 0, 0.2, 0.5, 1, 2, 3 and 4%) alloys is summarized in Figure 1. From Figure 1a, we can see that the alloy has α-Mg, α-Mn, Mg_2_Sn and MgZn_2_ phases. When 0.2%Al is added, Al element does not combine with other elements, and the alloy is still dominated by α-Mg, α-Mn, Mg_2_Sn and MgZn_2_ phases. When the content of Al increases to 0.5%, the Al_8_Mn_5_ phase is formed, and the content of Al increases further (1%), In addition, the Mg_32_(Al,Zn)_49_ phase and Al_11_Mn_5_ phase appear among the alloys, and when the Al content increases to 3%, the Al_2_Mg_5_Zn_2_ phase appears in the alloy.

**Figure 2:**
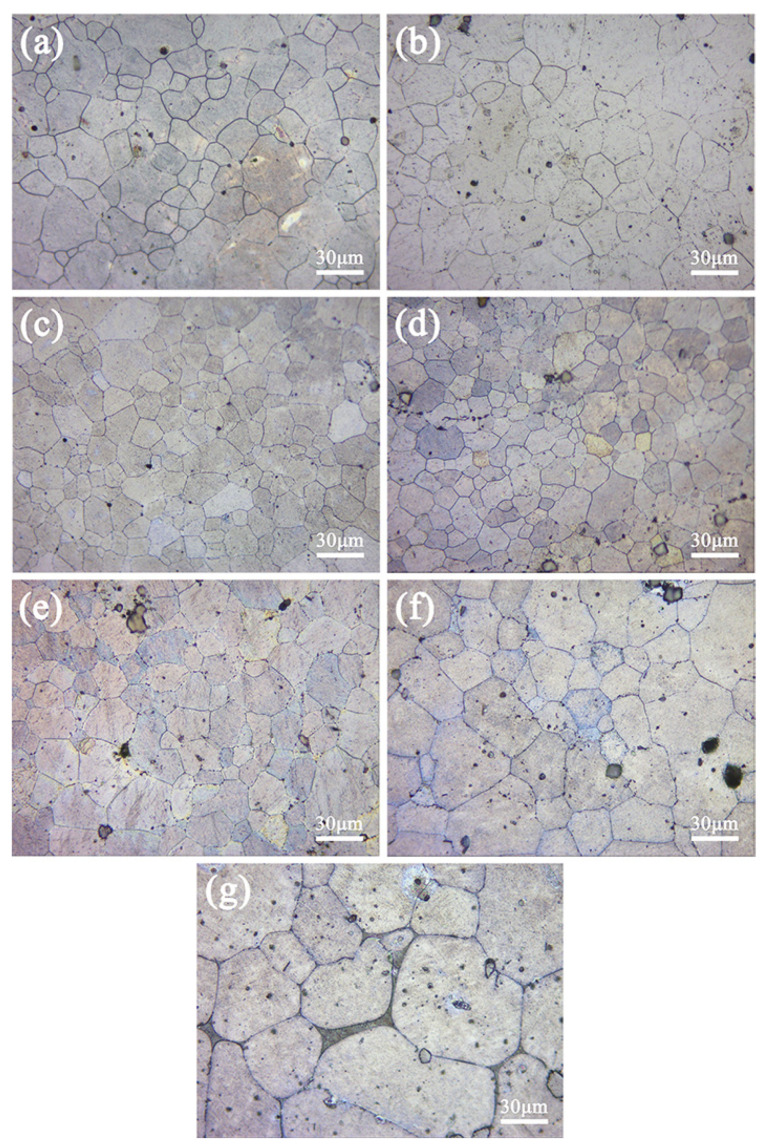
Optical micrographs of the single-aged ZTM641-xAl alloys, (**a**) x = 0, (**b**) x = 0.2, (**c**) x = 0.5, (**d**) x = 1, (**e**) x = 2, (**f**) x = 3 and (**g**) x = 4.

**Figure 3:**
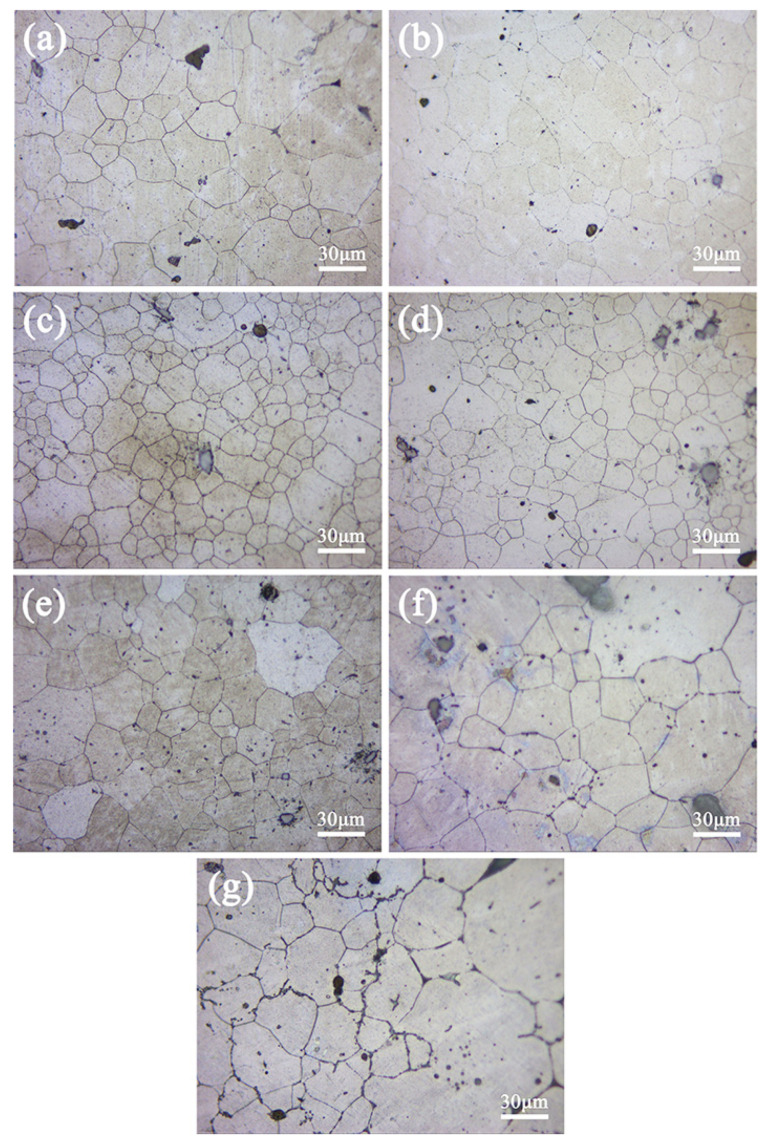
Optical micrographs of the double-aged ZTM641-xAl alloys, (**a**) x = 0, (**b**) x = 0.2, (**c**) x = 0.5, (**d**) x = 1, (**e**) x = 2, (**f**) x = 3 and (**g**) x = 4.

**Figure 4:**
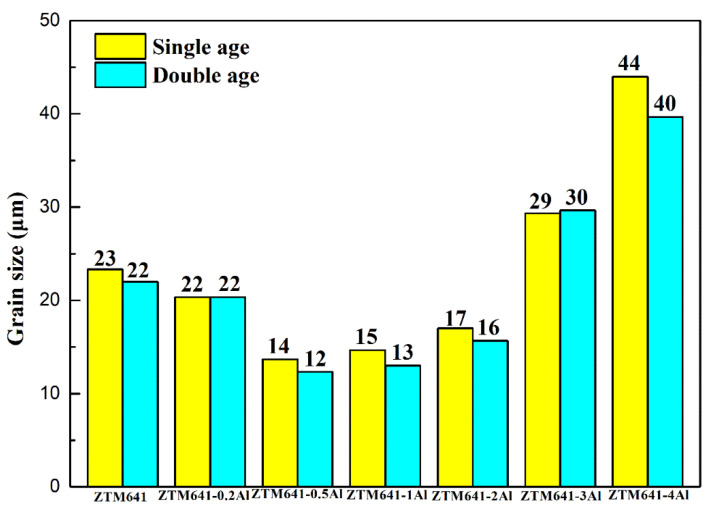
Average grain size of as-aged ZTM641-xAl alloys.

Figure 2, Figure 3 and Figure 4 show the microstructure and average grain size, respectively, of the aged ZTM641-xAl alloys. The average grain size is measured by the Heyn transversal method, the details can be seen in the last paper [23]. Comparing Figure 2 and Figure 3, it can be found that the microstructure of single aging and double aging is similar. The double-aged alloys are subjected to gradient treatment, and the time is longer, then the secondary grain growth is more adequate and the average grain size is slightly small, but not obvious. The ZTM641 alloy mainly comprises equiaxed crystals, but it is not uniform. The phases are basically distributed along the grain boundaries. When Al element is added, on the one hand, the volume fraction of phases slightly increases and the reveals that particles are mainly heat stable Mg_2_Sn and Al-Mn phases, as shown in the analysis results of Figure 1. On the other hand, when the Al content is 0.5%, the grains become more uniform, and the sizes are reduced to 12~14 μm (Figure 4). This can be ascribed to the evenly distributed Al content and the numerous dispersed fine particles that hinder the movement of grain boundary [10]. As Al content further increases to 2%, the alloys gradually change towards the mixed crystal structure, and the grain size increases. Compared with finer particles, this is due to the increasing coarse phases with a weaker dispersion, moreover, the impediment to grain growth is uneven, resulting in uneven grains and an increase in the average size [26]. In order to further verify the residual alloy phase, a SEM and an EDS analysis were conducted.

**Figure 5:**
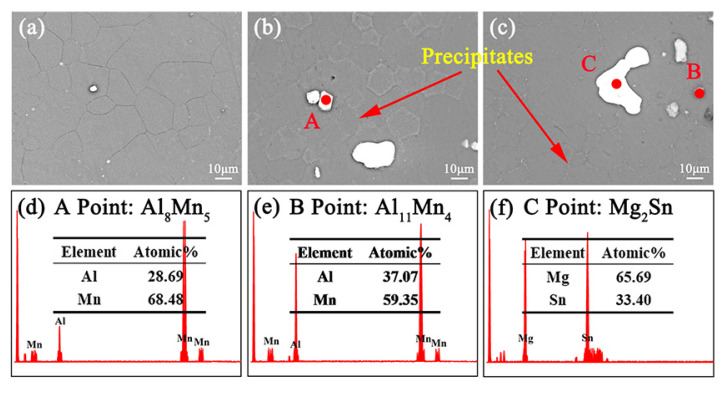
BSE-SEM micrographs of the single-aged ZTM641-xAl alloys, (**a**) x = 0, (**b**) x = 0.5, (**c**) x = 2, (**d**–**f**) corresponding EDS results of the points indicated in (**b**,**c**).

Figure 5 shows the backscatter electron (BSE) SEM microscope observations for the single-aged ZTM641-xAl alloy. The ZTM641 alloy exhibits a favorable aging result, and the remaining second phase comprises a bright Mg_2_Sn and a grey MgZn_2_ phase [25]. After adding Al content, on the one hand, it shows from Figure 5b that the dotted gray phase at point A is Al_8_Mn_5_. As the Al content increased, Al_8_Mn_5_ changes to Al_11_Mn_4_ phase, as shown at point B in Figure 5c, which is consistent with the report [27]. Additionally, Dispersed fine phases are found at the grain boundaries, and with increases in the Al element, the fine phase increases, and the phases are continuously distributed on grain boundaries. Previous research has shown that these phases are Mg-Zn phases [28]. This indicates that, with the addition of a small amount of the Al element, the fine and dispersed precipitated phases play the role of pinning the grain boundaries, which reduces the grain size. However, as the Al content further increases, the bulk phases’—such as the Al-Mn phases and Mg_2_Sn phases—volume fractions increase, and the phases located on the grain boundaries become continuous, resulting in an increase in the grain size, which is consistent with above discussion. Furthermore, the specific morphology and type of these phases cannot be clearly observed by SEM due to its lower resolution. Therefore, the TEM observation is adopted to identify the fine phases.

**Figure 6:**
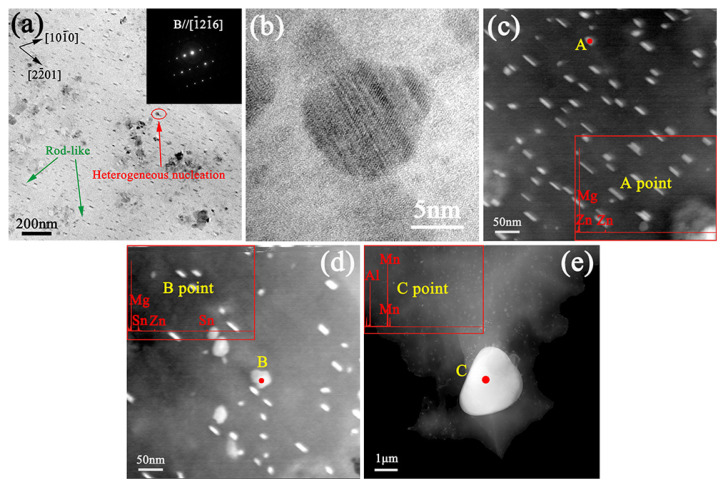
TEM micrographs of the single-aged ZTM641-1Al alloy, (**a**) Bright-field (BF) TEM image, taken along the [$\overset{-}{1}$2$\overset{-}{1}$6]_α-Mg_ zone axis, (**b**) HRTEM image of “T” phase (**c**–**e**) HAADF-STEM image, inset: corresponding EDS results of the points.

**Figure 7:**
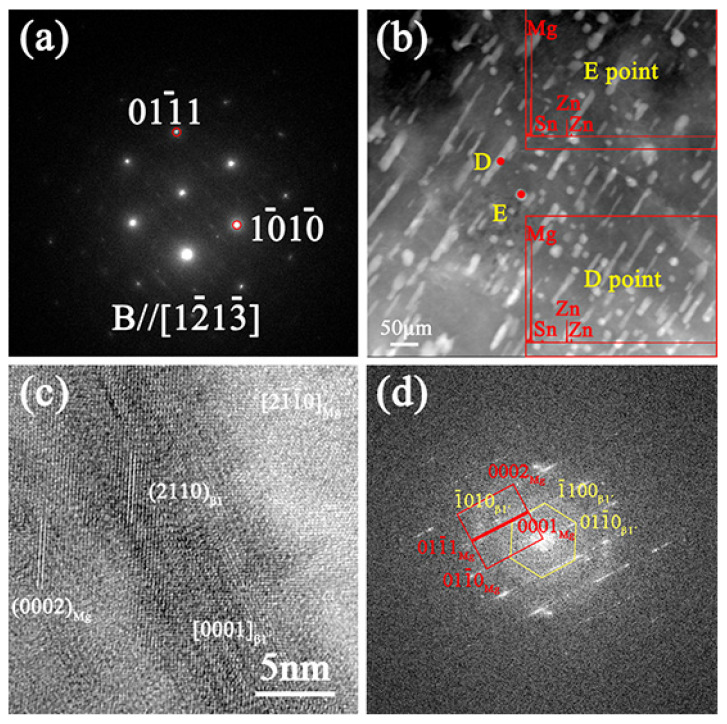
TEM micrographs of the single-aged ZTM641-1Al alloy, (**a**) Diffraction spots of the matrix, (**b**) HAADF-STEM, taken along the [$\overset{-}{1}$2$\overset{-}{1}$3]_α-Mg_ zone axis, inset: corresponding EDS results of the points (**c**) HRTEM image of the *β*_1_’-MgZn_2_ phase and (**d**) corresponding FFT pattern of (**c**).

Figure 6 and Figure 7 show the TEM images of single-aged ZTM641-1Al alloy. According to the incident beam parallel to [$\overset{-}{1}$2$\overset{-}{1}$6]_α-Mg_, Figure 6a shows the BF-TEM images subjected to single-aged treatment (180 °C/12 h). Three precipitates are dispersed in single-aged alloy: rod-shaped, disc-shaped and irregular phases. Among them, the rod-shaped precipitates account for the vast majority, and it can be obviously observed that rod-shaped precipitates have an obvious and consistent growth relationship between the matrix. It is reported that the precipitate perpendicular to the rod-shaped phase is the disc-shaped *β*_2_’-MgZn_2_ phase, this lays on the base surface, and its strengthening effect is much weaker than that of the rod-shaped phase [29]. In order to further verify the types of these fine phases, an EDS energy spectrum analysis is carried out. Based on the EDS data at point D in Figure 7, the rod-shaped precipitates are identified as *β*_1_’-MgZn_2_, and the EDS results at point A in Figure 6 and E in Figure 7 show that these precipitated phases perpendicular to the *β*_1_’are *β*_2_’, and point B in Figure 6 is irregular shaped Mg_2_Sn phase, which is consistent well with the above results. In addition, a micron-sized Al-Mn phase is also observed in Figure 6e. This is considered a residual phase, which is undissolved in the matrix during solid solution stage. The *β*_1_’ perpendicular to basal plane is the main strengthening phase, which can effectively pin the grain boundaries during alloy deformation process and prevent dislocation slipping [30].

According to the incident beam parallel to [$\overset{-}{1}$2$\overset{-}{1}$3]_α-Mg_, Figure 7b shows that a small amount of Sn is found. When the solute Sn atoms are concentrated at one end of *β*_1_’, the heterogeneous nucleation effect is greatly reduced, and due to the increased nucleation energy barrier, the Mg_2_Sn phase is easy to nucleate at one end of the rod to form a “T” shaped phase, resulting in the a shorter length than that of ordinary *β*_1_’ [31,32], which is consistent with the result in Figure 6b. Figure 7c is the HRTEM of *β*_1_’ in the single-aged treatment of ZTM641-1Al alloy. According to the FFT of *β*_1_’ in Figure 7d, it can be verified that the orientation relationship between *β*_1_’ and matrix is: [2$\overset{-}{1}$$\overset{-}{1}$0]_α-Mg_//[0001]${}_{{\beta 1}^{\text{’}}}$, (0002)_α-Mg_//($\overset{-}{2}$110)${}_{{\beta 1}^{\text{’}}}$ [33,34].

### 3.2. Mechanical Properties

#### 3.2.1. Mechanical Properties at Room Temperature

**Figure 8:**
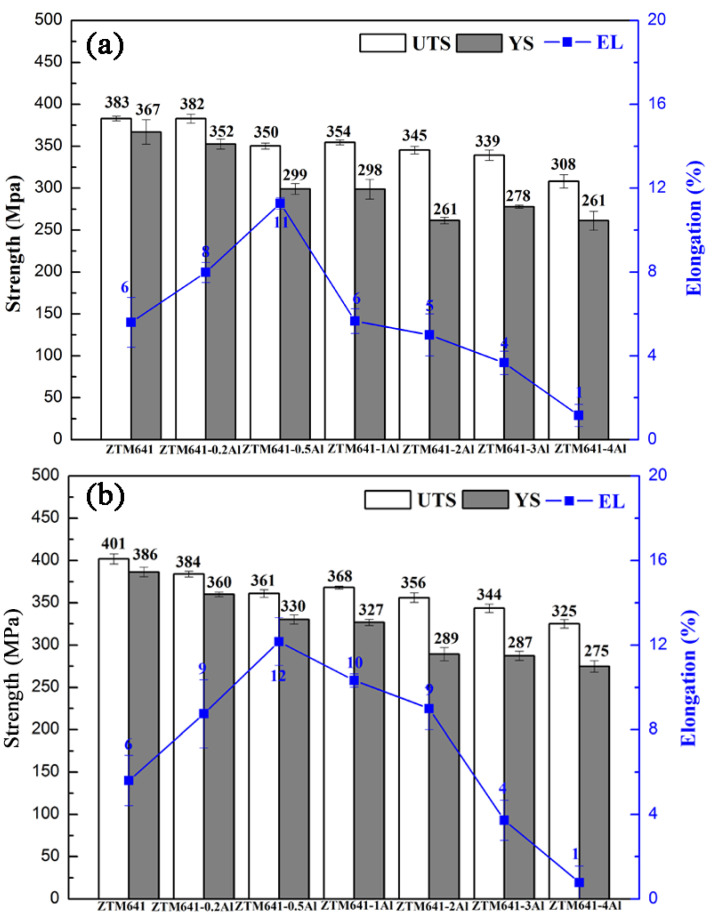
Mechanical properties of the as-aged ZTM641-xAl alloys at room temperature, (**a**) single age, (**b**) double age.

Figure 8 shows the mechanical properties of the as-aged ZTM641-xAl alloys at room temperature. The performances of double-aged alloys are better than those of the single-aged alloys. However, considering the time cost, single-aged treatment is appropriate for this series of alloys. In general, regardless of single-aged alloys or double-aged alloys, when Al element increases, the strength of as-aged alloys gradually decreases, and the elongation first increases while a further quantity decreases. Nie et al. [35], showed that phases and interface are important factors affecting the properties of alloys. Therefore, the properties are discussed from these two aspects. First, when the content of Al is 0.2%, no new phase is generated, and Al element is mainly dissolved in the matrix. On the one hand, according to the literature [36], Al element can effectively reduce the layer fault energy of the alloy, on the other hand, a part of Al element is gathered at the grain boundary, which can promote grain boundary slip and improve the plasticity of the alloy. However, Al atomic segregation is not conducive to the enhancement of strength. When the content of Al increases to 0.5%, the Al_8_Mn_5_ phase is gradually formed. At this time, the size of phases is small, and the distribution is more dispersed, in which the small precipitated phases dispersed within the grain can play the role of pinning dislocation, slowing down the rate of dislocation accumulation at the grain boundary during material deformation, and improving the storage capacity of dislocation within the material. When the alloy is deformed, it may start more slip systems and improve the elongation. Moreover, according to the previous microstructure analysis (Figure 4), the grain size of ZTM641-0.5Al alloy is the smallest. When the grain size is smaller, the smaller force shared by each grain, the easier movement between them, and the more conducive they are to plastic de-formation. This is the reason why the ZTM641-0.5Al alloy has the best elongation. When the content of Al increases to 1%, the Mg_32_(Al,Zn)_49_ phase and Al_11_Mn_5_ phase appear in the alloy, and the number of precipitated phases increases. When Al element continues to rise to 3%, the Al_2_Mg_5_Zn_2_ phase appears. It is worth noting that the Al_11_Mn_5_ phase, Mg_32_(Al,Zn)_49_ and Al_2_Mg_5_Zn_2_ phases are all brittle phases, the large brittle phases can easily to become a crack source during the tensile process, so their appearance will deteriorate the properties of alloys. Furthermore, when Al content increases, the increasingly precipitated phases are distributed on the matrix and grain boundaries, resulting in the cleavage of the matrix, which reduces the coordinated deformation ability, then, the plasticity of alloys significantly deteriorates. Combined with strength and plasticity, the double-aged ZTM641-0.2Al exhibits favorable mechanical properties at room temperature, the UTS, YS and elongation are 384 MPa, 360 MPa and 9%, respectively.

**Figure 9:**
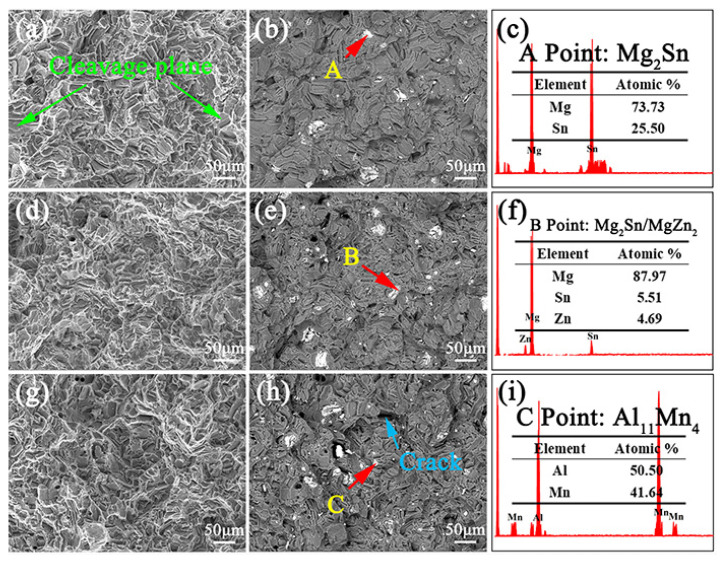
(**a**,**d**,**g**) SE-SEM, (**b**,**e**,**h**) BSE-SEM micrographs of fracture surface of the single-aged ZTM641-xAl alloys tested at room temperature, (**a**,**b**) x = 0, (**d**,**e**) x = 0.5, (**g**,**h**) x = 2, (**c**,**f**,**i**) corresponding EDS results of the points indicated in (**b**,**e**,**h**).

Figure 9 shows the secondary electron (SE) and BSE-SEM microscope observations of single-aged ZTM641, ZTM641-0.5Al and ZTM641-2Al alloys at room temperature. From Figure 9a,d,g, we can find cleavage planes, dimples, tear edges and granular bumps in fracture surface, and these characteristics are suggestive of mixed fracture. For ZTM641 alloy, many cleavage planes are generated in coarse grains, as shown in Figure 9a, which is consistent with Figure 2b. The quantity and size of the cleavage planes decreases with a small quantity of Al element. However, the fracture mode of ZTM641-2Al alloy is not changed significantly. From BSE-SEM images, combined with the EDS results, the phase of the white color is Mg_2_Sn, white and gray is a mixed compound of MgZn_2_ and Mg_2_Sn, and gray bulk phase is Al_11_Mn_4_, which is consistent with above results and previous reports [37]. For ZTM641-2Al alloy, the number of phases slightly increases, and cracks appear in some coarse phases. The mixed phases greatly reduce the bonding force between the matrix, and cracks are easily formed during deformation process. Moreover, according to previous research [17,20,38], on the one hand, the α-Mn phase can become nucleation cores of strengthening *β*_1_’ phase in as-aged treatment process, but when Al content increases, Mn combines with Al element to form Al-Mn phases. On the other hand, the purification effect of Mn decreases with a high amount of the Al element, which increases the possibility of alloy defects. Consequently, the beneficial effects of Al content are covered by the above adverse effects for high Al-containing alloys, which leads to worse mechanical properties.

**Figure 10:**
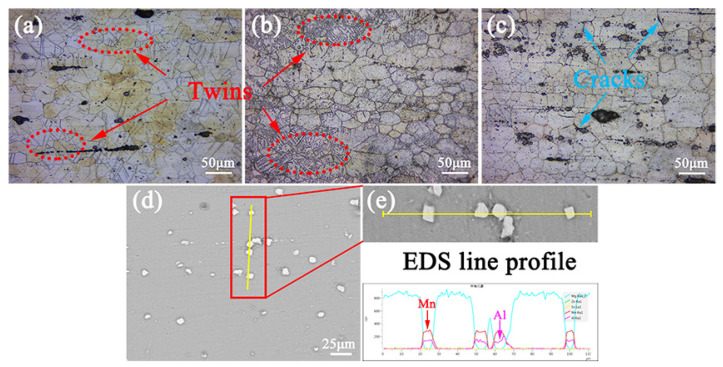
Optical images (**a**–**c**) and SE-SEM (**d**) from longitudinal sections of the single-aged ZTM641-xAl alloys adjacent fracture surface tested at room temperature, (**a**) x = 0, (**b**) x = 0.5, (**c**,**d**) x = 2 alloy and (**e**) EDS line profile in (**d**).

Figure 10 shows the images of single-aged ZTM641-xAl alloys tested at room temperature. For ZTM641 alloy, a large number of twins are found. When Al content increases, the twin density first increases and then decreases. The grain size exhibits opposite phenomenon: the number of phases gradually increases, which is consistent with Figure 2. Since twins have the effect of coordinating deformation, the decrease in the number of twins indicates that the alloy exhibits worse elongation. Furthermore, for ZTM641-2Al alloy, we can clearly see that the bulk phases are mainly distributed on grain boundary, and cracks mainly occur here, which indicates that the crack may first occur from the stress concentration caused by bulk phases, resulting in the fracture of the alloy. According to the EDS line profile result from Figure 10e, the bulk phases mainly contain Al and Mn elements, combined with Figure 9i, which can be determined as the Al-Mn phase. This indicates that excessive Al-Mn phases have a poor effect on properties.

#### 3.2.2. Mechanical Properties at Elevated Temperature

**Figure 11:**
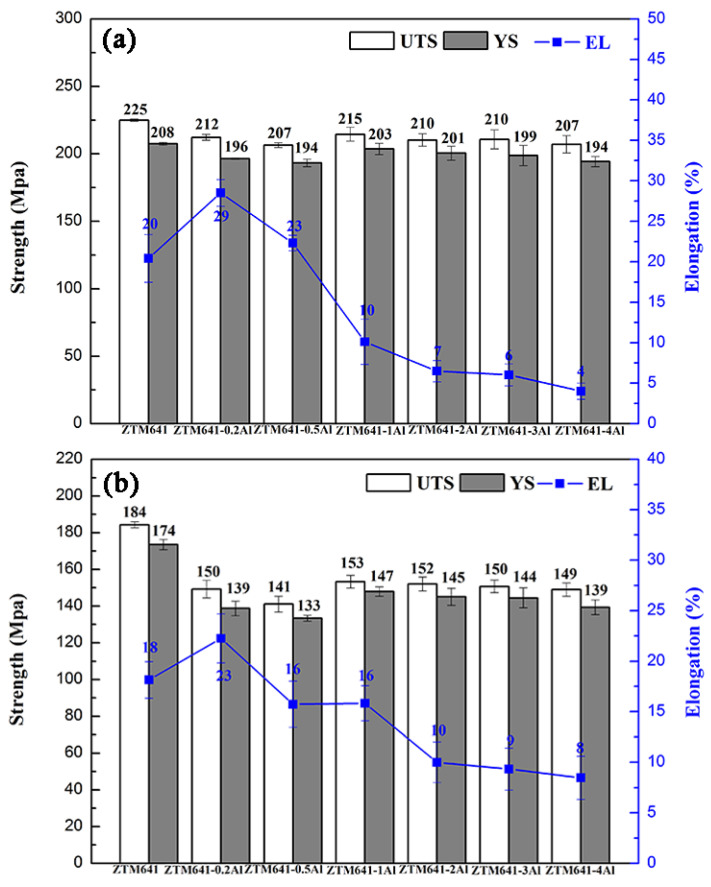
Mechanical properties of the double-aged ZTM641-xAl alloys tested at different temperatures, (**a**) 150 °C and (**b**) 200 °C.

**Figure 12:**
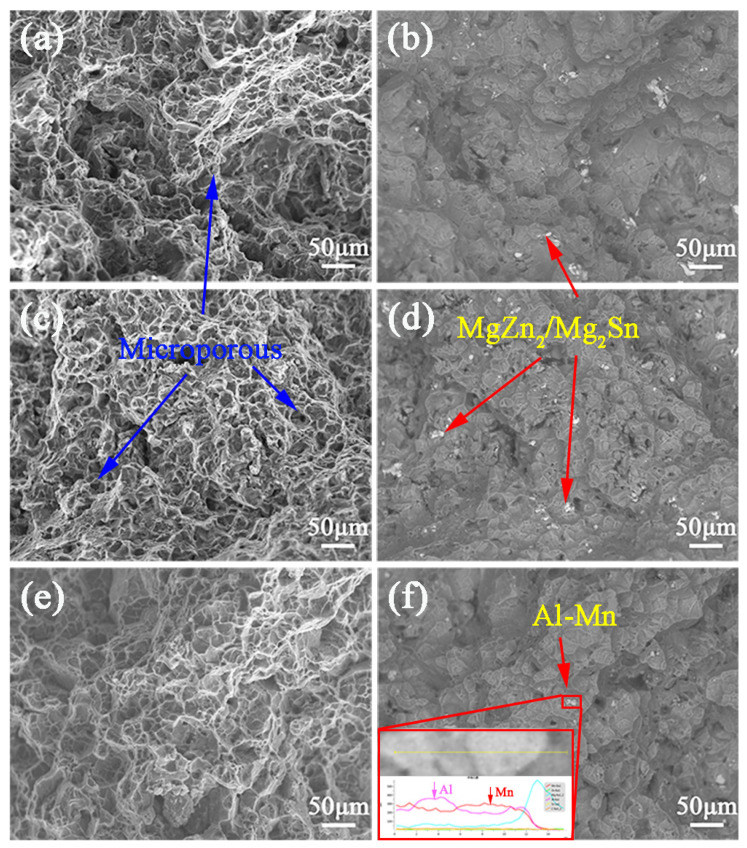
(**a**,**c**,**e**) SE-SEM, (**b**,**d**,**f**) and BSE-SEM micrographs of the fracture surface of double-aged ZTM641-xAl alloys tested at 200 °C, (**a**,**b**) x = 0, (**c**,**d**) x = 0.2 and (**e**,**f**) x = 2.

Figure 11 shows the mechanical properties of double-aged ZTM641-xAl (x = 0, 0.2, 0.5, 1, 2, 3 and 4%) alloys at 150 °C and 200 °C. We can see from Figure that the mechanical properties are obviously affected by temperature. It is obvious that the properties of alloys at 150 °C are generally better than those at 200 °C. Further, when the tensile tests are carried out at 150 °C, from Figure 11a, we can find that the elongation is improved by a small amount of Al. The ZTM641-0.2Al alloy has the highest elongation. According to the analysis in Figure 8, we know that when 0.2%Al is added to the alloy, Al element can reduce the layer fault energy of the alloy and improve the plasticity. When the deformation temperature increases (150, 200 °C), the amplitude of thermal vibration of all atoms—including Al—increases, the Critical Resolved Shear Stress of non-base slip decreases, and a variety of slip systems start to participate in the coordinated deformation, which greatly improves the high-temperature plasticity of the alloy. When Al element is added to 0.5%, it can be seen from the previous analysis that its combination with Mn element generates Al_8_Mn_5_ phase with a small particle size. The fine, precipitated phase dispersed in grains can play the role of pinning dislocation and slow the accumulation rate of dislocation at the grain boundary during material deformation. However, when the deformation is performed at a higher temperature (150, 200 °C), the activation energy of atoms decreases, the activity of atoms is higher, and the hindrance effect of the tiny precipitated phase is reduced, which causes the dislocations stored in the original materials to relax, further, deformation at this temperature is not conducive to the operation of a greater number of slip systems, and the properties of the alloy are reduced. When Al element increases further, as can be seen from Figure 1, the Mg_32_(Al,Zn)_49_ phase, Al_11_Mn_5_ phase and Al_2_Mg_5_Zn_2_ phase appear successively. As mentioned above, the Al-Mn phase, Mg_32_(Al,Zn)_49_ phase and Al_2_Mg_5_Zn_2_ phase are all brittle phases. Due to the high content of Al, the particle size of precipitated phase is large, which is not conducive to coordinated deformation, but can also easily become a crack source and reduce the plasticity of the alloy. It is worth noting that these phases are also thermally stable phases. Among them, the melting points of Mg_32_(Al,Zn)_49_ phase and Al_11_Mn_5_ phase are 535 °C and 393 °C, respectively. When the alloy is stressed and deformed at a high temperature, due to the high melting point, these precipitated phases are not easy to soften, which can nail grain boundaries, hinder dislocation slip and improve the strengths. The nailing effect satisfies the Zener formula [39]:

(1) $$F_{Z}=\frac{3f\gamma }{2r}$$

where f is the volume fraction of the phase, γ is the grain boundary energy and r is the average size of the phase. As can be seen from the formula, the size of phase is smaller and the number is larger, and the nailing effect is more obvious. Among these alloys (ZTM641-xAl, x = 1, 2, 3, 4), the size of the precipitated phases of ZTM641-1Al is relatively small, so the strengthening effect is the best. In addition, the decreasing trend of strength is smoother than that of room temperature. The reason for this may be the mutual effect of the decreasing difference between strong plane and secondary dense row, as well as the decreasing volume fraction of bulk phases compared with room temperature, which is further explained in Figure 12 [40]. The alloy with the best combination of strength and elongation is ZTM641-0.2Al alloy, the UTS, YS and elongation are 212 MPa, 196 MPa and 29%, respectively. Compared with ZTM641 alloy, the strength of this alloy is slightly worse, but the elongation is relatively higher. When the tensile tests are carried out at 200 °C, the strength variation tendency of mechanical properties is similar to that at 150 °C. The ZTM641-0.2Al alloy exhibits comprehensive mechanical properties, the UTS, YS and elongation are 150 MPa, 139 MPa and 23%, respectively.

Figure 12 shows the SE and BSE-SEM microscope observations for double-aged ZTM641, ZTM641-0.2Al and ZTM641-2Al alloys at 200 °C. For ZTM641 alloy, the fracture of the alloy is featured by cleavage plane, microporous, tear edges and granular bumps, which are the characteristics of mixed fracture. For ZTM641-0.2Al, the cleavage plane can hardly be seen, and it is confirmed as ductile fracture. Through the BSE image and EDS data in Figure 12d, the white and gray colors are mainly a mixed compound of MgZn_2_ and Mg_2_Sn. When Al element reaches 2%, as shown in Figure 12e, the fracture morphology is similar to that of Figure 12a, indicating that the fracture mode of ZTM641-2Al alloy is also mixed fracture. By observing Figure 12f, combined with energy spectrum line scanning, it is found that the dotted gray phase is mainly Al and Mn elements, and it can be concluded that this is the Al-Mn phase.

**Figure 13:**
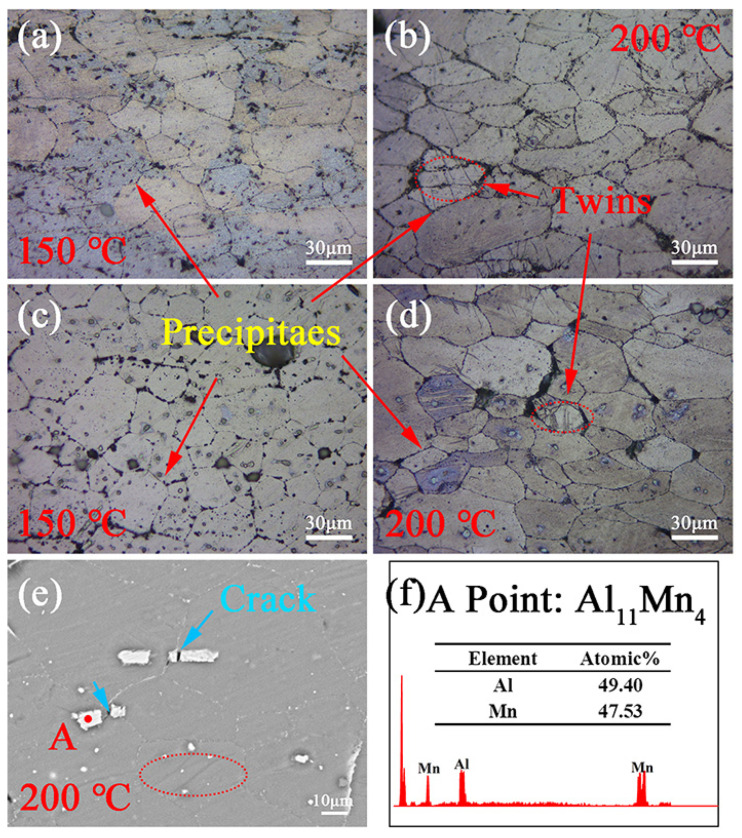
Optical images (**a**–**d**) and BSE-SEM (**e**) from longitudinal sections adjacent to fracture surface tested at 150 °C (**a**,**c**) and 200 °C (**b**,**d**,**e**) of the double-aged ZTM641-xAl alloys, (**a**,**b**) x = 0.2, (**c**–**e**) x = 2, (**f**) corresponding EDS result of the point indicated in (**e**).

Figure 13 shows the optical images and BSE-SEM of double-aged ZTM641-0.2Al and ZTM641-2Al alloys at 150 °C and 200 °C. For 150 °C, a lot of discontinuity precipitates in ZTM641-0.2Al alloy are distributed along grain boundaries and in grains. In comparison, the ZTM641-2Al alloy contains larger sized and a greater number of precipitates distributed along the grain boundaries. This indicates that stress concentration and crack are likely to occur along the grain boundaries, resulting in worse elongation, which is consistent with mechanical properties. At 200 °C, the number of precipitates within grains is significantly reduced, and the precipitates along the grain boundaries become continuous. Combined with above analysis in Figure 5 and Figure 12f, the bulk phases are Al-Mn. In a word, for ZTM641-2Al alloy, the continuous distribution of the precipitates at grain boundary and increasing temperature leads to the weakening of inter-grain bonding force, leading to worse elongation with increasing temperature, as is consistent with the properties. Moreover, although twins are found in the ZTM641-2Al alloy, the above-mentioned adverse effects on elongation are not eliminated. On the contrary, for ZTM641-0.2Al alloy, with the increasing temperature, the size of the precipitates become finer, and the twins are beneficial to the coordinated deformation, which leads to a certain degree of improvement in the elongation.

## 4. Conclusions

The effects of Al addition (0, 0.2, 0.5, 1, 2, 3 and 4%) on the microstructure and mechanical properties of the aged ZTM641 alloy were systematically investigated in this paper. The conclusions can be summarized as follows:

1. There is a difference between the single-aged and double-aged microstructure. The aged ZTM641 alloy mainly consists of α-Mg, α-Mn, MgZn_2_ and Mg_2_Sn phases. The Al element mainly forms Al_8_Mn_5_, Al_11_Mn_4_, Al_2_Mg_5_Zn_2_ and Mg_32_(Al,Zn)_49_ phases. With increases in the addition of Al, the average grain size first decreases and then increases, and the undissolved phases increase. The average grain size of ZTM641-0.5Al alloy is the smallest, and the single-aged and double-aged grain sizes are 14 μm and 12 μm, respectively.
2. The mechanical properties of double-aged alloys are superior to single-aged alloys. The Al element can improve the comprehensive properties at room temperature, 150 °C and 200 °C. The double-aged ZTM641-0.2Al alloy exhibits favorable mechanical properties at room temperature, the UTS, YS and elongation are 384 MPa, 360 MPa and 9%, respectively. Furthermore, the double-aged ZTM641-0.2Al alloy exhibits comprehensive mechanical properties at 150 °C, that is, the UTS, YS and elongation are 212 MPa, 196 MPa and 29%, respectively, which is about 45% higher than that of elongation of ZTM641.
3. At room temperature, the ZTM641-xAl alloys exhibit mixed fracture. At 200 °C, with increases in the Al content, the fracture mechanisms of alloys are mixed fracture, ductile fracture and mixed fracture, respectively. Furthermore, the elongation of ZTM641-xAl (x ≤ 0.5%) decreases when the temperature rises from 150 °C to 200 °C, which is mainly due to coarse phases at the grain boundaries that can easily cause microcracks.

## Data Availability

All data are available from the corresponding author upon reasonable request.

## References

[1] Agnew S.R., Nie J.F. (2010). Preface to the viewpoint set on: The current state of magnesium alloy science and technology. Scr. Mater..

[2] Hono K., Mendis C.L., Sasaki T.T., Oh-ishi K. (2010). Towards the development of heat-treatable high-strength wrought Mg alloys. Scr. Mater..

[3] Wang J.F., Gao S., Song P.F., Huang X.F., Shi Z.Z., Pan F.S. (2011). Effects of phase composition on the mechanical properties and damping capacities of as-extruded Mg–Zn–Y–Zr alloys. J. Alloy. Compd..

[4] Kaya S., Yılan F., Urtekin L. (2022). Influences of Cr on the microstructural, wear and mechanical performance of high-chromium white cast iron grinding balls. J. Mater. Manuf..

[5] Coban H., Koklu U. (2022). Drilling of AZ31 Magnesium Alloy under dry and cryogenic conditions. J. Mater. Manuf..

[6] Gao L., Yan H., Luo J., Luo A.A., Chen R.S. (2013). Microstructure and mechanical properties of a high ductility Mg–Zn–Mn–Ce magnesium alloy. J. Magnes. Alloy..

[7] He X., Chen J.H., Yan H.G., Su B., Zhang G.H., Miao C.M. (2013). Effects of minor Sr addition on microstructure and mechanical properties of the as-cast Mg-4.5Zn-4.5Sn-2Al-based alloy system. J. Alloy. Compd..

[8] Yang H.G., Li Y.B., Cui H.Z. (2012). High-temperature creep behavior of Mg-9Gd-4Y-0.5Zr alloy. Heat Treat. Met..

[9] Gao X., Nie J.F. (2007). Structure and thermal stability of primary intermetallic particles in an Mg-Zn casting alloy. Scr. Mater..

[10] Oh-ishi K., Hono K., Shin K.S. (2008). Effect of pre-aging and Al addition on age-hardening and microstructure in Mg-6wt% Zn alloys. Mater. Sci. Eng. A.

[11] Doan J.P., Ansel G. (2020). Some effects of zirconium on extrusion properties of magnesium-base alloys containing zinc. Trans. AIME.

[12] Wang B., Pan F.S., Chen X.H., Guo W., Mao J.J. (2016). Microstructure and mechanical properties of as-extruded and as-aged Mg–Zn–Al–Sn alloys. Mater. Sci. Eng. A.

[13] Jia Z., Yu Y.Z., Yu B., Fu L., Hu W.Y., Shao Y.C. (2022). Effect of Ca and Zr additions on microstructure and mechanical properties of as-extruded Mg-3Sn alloy. Materials.

[14] Mendis C.L., Bettles C.J., Gibson M.A., Hutchinson C.R. (2006). An enhanced age hardening response in Mg–Sn based alloys containing Zn. Mater. Sci. Eng. A.

[15] Zhang J., Li Z.S., Guo Z.X., Pan F.S. (2006). Solidification microstructural constituent and its crystallographic morphology of permanent-mould-cast Mg-Zn-Al alloys. Trans. Nonferrous Met. Soc. China.

[16] Wan X.F., Ni H.J., Huang M.Y., Zhang H.L., Sun J.H. (2013). Microstructure, mechanical properties and creep resistance of Mg-(8%–12%)Zn-(2%–6%) Al alloys. Trans. Nonferrous Met. Soc. China.

[17] Shi G.L., Zhang D.F., Zhao X.B., Zhang K., Li X.G., Li Y.J., Ma M.L. (2013). Precipitate evolution in Mg-6 wt%Zn-1 wt%Mn alloy. Rare Met. Mater. Eng..

[18] Yin D.S., Zhang E.L., Zeng S.Y. (2008). Effect of Zn on mechanical property and corrosion property of extruded Mg-Zn-Mn alloy. Trans. Nonferrous Met. Soc. China.

[19] Wang Q.H., Wang L., Zhai H.W., Chen Y., Chen S. (2022). Establishment of constitutive model and analysis of dynamic recrystallization kinetics of Mg-Bi-Ca Alloy during hot deformation. Materials.

[20] Hou C.H., Qi F.G., Ye Z.S., Zhao N., Zhang D.F., Ouyang X.P. (2020). Effects of Mn addition on the microstructure and mechanical properties of Mg–Zn–Sn alloys. Mater. Sci. Eng. A.

[21] Kim S.H., Lee J.U., Ye J.K., Jung J.G., Park S.H. (2018). Controlling the microstructure and improving the tensile properties of extruded Mg-Sn-Zn alloy through Al addition. J. Alloy. Compd..

[22] Chen X., Li S.L., Wan Y.C. (2022). The effect of initial grain size on the nanocrystallization of aZ31 Mg alloy during rotary swaging. Materials.

[23] Hou C.H., Ye Z.S., Qi F.G., Wang Q., Li L.H., Ouyang X.P., Zhao N. (2021). Effect of Al addition on microstructure and mechanical properties of Mg−Zn−Sn−Mn alloy. Trans. Nonferrous Met. Soc. China.

[24] Hu G.S., Zhang D.F., Dong Y.F., Chen X., Jiang L.Y., Pan F.S. (2015). Microstructures and mechanical properties of as-extruded and heat treated Mg–6Zn–1Mn–4Sn–1.5Nd alloy. Trans. Nonferrous Met. Soc. China.

[25] Qi F.G., Zhang D.F., Zhang X.H., Xu X.X. (2014). Effect of Sn addition on the microstructure and mechanical properties of Mg–6Zn–1Mn (wt.%) alloy. J. Alloy. Compd..

[26] Song X.Y., Gu N.J., Liu G.Q., Wang B.Q. (2000). Computer simulation of the influence of second-phase particle quantity on matrix grain growth. Acta Metall. Sin..

[27] Yu Z.W., Hu M.D., Tang A.T., Wu M.S., He J.J., Gao Z.Y., Wang F.Y., Li C.Y., Chen B., Liu J.G. (2017). Effect of aluminium on the microstructure and mechanical properties of as-cast magnesium–manganese alloys. Mater. Sci. Technol..

[28] Qi F.G., Luo W.Z., Wu C.P., Zhao N., Ye Z.S., Hou C.H., Zhang D.F. (2020). Microstructure evolution and mechanical properties of ZM81-xSn wrought magnesium alloys. Rare Met. Mater. Eng..

[29] Wei L.Y., Dunlop G.L., Westengen H. (1995). The intergranular microstructure of cast Mg-Zn and Mg-Zn-rare earth alloys. Metall. Mater. Trans. A-Phys. Metall. Mater. Sci..

[30] Gao X., Nie J.F. (2007). Characterization of strengthening precipitate phases in a Mg–Zn alloy. Scr. Mater..

[31] Gorny A., Katsman A. (2008). Precipitation- and stress-influenced coarsening in Mg-based Mg–Zn–Sn–Y and Mg–Zn–Sn–Sb alloys. J. Mater. Res..

[32] Gorny A., Katsman A., Shepelev D. (2006). Precipitation sequence in Mg-Zn-Sn based alloys. Mater. Res. Soc. Symp. Proc..

[33] Clark J.B. (1965). Transmission electron microscopy study of age hardening in a Mg-5 wt.% Zn alloy. Acta Metall..

[34] Chun J., Byrne J. (1969). Precipitate strengthening mechanisms in magnesium zinc alloy single crystals. J. Mater. Sci..

[35] Nie J.F. (2012). Precipitation and hardening in magnesium alloys. Metall. Mater. Trans. A.

[36] Zhang J., Dou Y.C., Dong H.B. (2014). Intrinsic ductility of Mg-based binary alloys: A first-principles study. Scr. Mater..

[37] Cohen S., Goren-Muginstein G., Avraham S., Rashkova B., Dehm G. (2005). Precipitation hardening in Mg-Z-Sn alloys with minor additions of Ca and Si. Z. Für Met..

[38] Nam N.D. (2014). Corrosion behavior of Mg–5Al based magnesium alloy with 1 wt.% Sn, Mn and Zn additions in 3.5 wt.% NaCl solution. J. Magnes. Alloy..

[39] Smith C.S. (1948). Grains, phases, and interfaces an interpretation of microstructure. Met. Technol..

[40] Xiao Y., Zhang X.M., Chen J.M., Jiang H. (2006). Microstructures and mechanical properties of extruded Mg-9Gd-4Y-0.6Zr-T5 at elevated temperatures. Trans. Nonferrous Met. Soc. China.

